# Retrospective chart review of cases of steroid-responsive catatonia: exploring a potential autoimmune aetiology

**DOI:** 10.1192/bjo.2025.10806

**Published:** 2025-08-15

**Authors:** Rifat Serav Ilhan, Jonathan P. Rogers, Kazım Cihan Can, Berker Duman, Burçin Çolak, Meram Can Saka, Seyda Erdoğan, Rezzak Yilmaz, Canan Yucesan, Mine Araz, Güle Çınar, Sena Ünal, Özlem Doğan, Emine Uslu, Thomas A. Pollak

**Affiliations:** Department of Psychiatry, Ankara University Faculty of Medicine, Ankara, Turkey; Division of Psychiatry, University College London, London, UK; Department of Neurology, Ankara University Faculty of Medicine, Ankara, Turkey; Department of Nuclear Medicine, Ankara University Faculty of Medicine, Ankara, Turkey; Department of Clinical Microbiology and Infectious Diseases, Ankara University Faculty of Medicine, Ankara, Turkey; Department of Radiology, Ankara University Faculty of Medicine, Ankara, Turkey; Department of Medical Biochemistry, Ankara University Faculty of Medicine, Ankara, Turkey; Department of Rheumatology, Ankara University Faculty of Medicine, Ankara, Turkey; Section of Neuropsychiatry, Department of Psychosis Studies, King’s College London, London, UK

**Keywords:** Catatonia, autoimmune encephalitis, autoimmune psychosis

## Abstract

**Background:**

Catatonia, a neuropsychiatric syndrome, can be associated with inflammatory conditions of the central nervous system.

**Aims:**

To explore steroid-responsive catatonia with possible autoimmune origins.

**Method:**

A retrospective investigation was conducted of clinical and paraclinical features, including imagining, serum, and cerebrospinal fluid findings in ten patients presenting with subacute onset catatonia and treated with steroid pulse therapy between January 2022 and January 2024.

**Results:**

A retrospective chart review identified ten patients (of a total of 56) with steroid-responsive subacute onset catatonia. Catatonia types varied. All patients were positive for delirium and psychotic symptoms. Imaging and cerebrospinal fluid results indicated non-specific signs of central nervous system inflammation. Intravenous 1 g methylprednisolone pulse therapy resulted in complete remission in all patients. Autoantibodies for limbic and paraneoplastic encephalitis were negative for all patients. None of the patients fulfilled the criteria for definite autoimmune encephalitis or autoantibody-negative probable autoimmune encephalitis.

**Conclusions:**

Diagnosis of autoimmune catatonia is challenging without autoantibody markers, but steroid responsiveness, combined with clinical and paraclinical features, may suggest an autoimmune mechanism.

Catatonia is a severe neuropsychiatric disorder characterised by movement abnormalities and disturbances in volition, autonomic function and affect. Medical causes are responsible for 20% of catatonia cases in populations that are not specifically chosen or selected. Autoimmune encephalitis is the most commonly reported inflammatory disease of the central nervous system (CNS).^1,2^

Recent reports indicate that anti-NMDAR (*N*-methyl-D-aspartate receptor) encephalitis is the primary autoimmune CNS disease that causes catatonia, representing 72% of cases.^2^ Other autoimmune disorders, including Hashimoto’s encephalopathy, neuropsychiatric systemic lupus erythematosus, antiphospholipid antibody syndrome and Sjogren’s syndrome, have also been identified as potential causes. Extensive diagnostic workups for autoimmune conditions in patients presenting with catatonia can lead clinicians to prompt targeted aetiological treatments such as steroids and other immunosuppressive agents.^2^

Here, we present ten cases involving various presentations of catatonic syndrome, resembling primary psychiatric disorders yet achieving complete remission with steroid treatment. We discuss the differential diagnosis process and management of these cases, emphasising the prompt administration of immunotherapy.

## Method

This retrospective chart review included patients admitted to Ankara University Adult Psychiatry Clinic between 2022 and 2024 who exhibited subacute-onset catatonia without direct evidence of an underlying autoimmune condition and were examined and treated prospectively with empirical steroid therapy. Medical investigations and treatment decisions were made at the time of admission to hospital, whereas data collection and analysis were conducted retrospectively to evaluate the clinical relevance of steroid responsiveness in suspected autoimmune catatonia cases. The data were obtained from existing medical records, including clinical assessments, laboratory results, neuroimaging findings and treatment responses documented during hospital treatment. In early 2022, the Neuropsychiatry Unit within the Department of Psychiatry initiated a standardised and prospective evaluation protocol for all patients presenting with a suspected autoimmune aetiology, including subacute-onset catatonia. Under this protocol, each patient underwent a comprehensive clinical and paraclinical assessment, which included structured cognitive testing, routine lumbar puncture and an extensive neurodiagnostic workup, all conducted in accordance with the most recent consensus guidelines regarding autoimmunity. By contrast, before 2022, standardised data collection, including lumbar punctures, structured encephalitis criteria assessments and follow-up cognitive testing, was not consistently performed or documented, rendering earlier cases unsuitable for inclusion in this study owing to insufficient data.

In this review, we aimed to identify and characterise a subset of catatonia patients who demonstrated a clinical response to steroids despite the absence of confirmatory autoantibody markers or a definitive autoimmune encephalitis diagnosis. A total of 56 patients admitted to hospital for subacute-onset catatonia between 2022 and 2024 at Ankara University Adult Psychiatry Clinic were initially screened. Ten of these patients received empirical steroid therapy on the basis of suspected autoimmunity and were included in the retrospective analysis. The remaining 46 patients who did not receive steroids were excluded from this study. Inclusion criteria were subacute-onset catatonia without a definitive autoimmune or neurodegenerative diagnosis at presentation, empirical intravenous (i.v.) methylprednisolone therapy (1 g per day for 5–7 days) owing to suspected autoimmunity, and comprehensive clinical and paraclinical evaluation performed during hospital treatment. Patients who did not receive empirical immunotherapy or were later diagnosed with a primer psychiatric or neurodegenerative disorder were excluded (*N* = 46). The diagnoses for these 46 patients included: dementia syndromes (*n* = 20), COVID-19–related encephalopathy (*n* = 2), medication-related catatonia (specifically lithium intoxication (*n* = 2), levetiracetam-induced (*n* = 2) and clozapine withdrawal (*n* = 2); total *n* = 6), substance-induced catatonia (*n* = 2), subarachnoid haemorrhage (*n* = 1), Wernicke encephalopathy (*n* = 3), glioblastoma multiforme (*n* = 1), neurodevelopmental disorders (e.g. autism spectrum disorder (*n* = 8)) and bipolar disorder (*n* = 3). Dementia syndromes were present in 20 patients; of these patients, 11 were already in follow-up for previously diagnosed neurodegenerative diseases, whereas nine received new diagnoses during the comprehensive medical evaluation process: these included *C9orf72*-mutation-associated frontotemporal dementia (*n* = 1), posterior-variant Alzheimer’s disease (*n* = 1), normal-pressure hydrocephalus (*n* = 1), antiphospholipid-antibody-associated vascular dementia/Noah syndrome (*n* = 1), CADASIL (*n* = 1), sporadic frontotemporal dementia (*n* = 2) and Alzheimer’s disease (*n* = 2).

A retrospective evaluation was conducted using electronic medical records and patient charts, focusing on clinical and paraclinical findings and treatment details for the ten patients included in the study. Clinical assessments extracted from medical records and patient charts included comprehensive psychiatric and neurological examination, the Bush–Francis Catatonia Rating Scale (BFCRS) for catatonia severity,^3^ the original version of the Confusion Assessment Method for delirium assessment (a four-item diagnostic algorithm for delirium)^4^ and the Neuropsychiatric Inventory Questionnaire. Laboratory and imaging data were obtained from the patients’ existing hospital records. Laboratory tests included full blood count, liver and kidney function tests, thyroid function tests, serum electrolytes, and markers such as vitamin B12, vitamin D, C-reactive protein and creatinine phosphokinase. Infectious markers were checked for pathogens, including *Toxoplasma gondii*, brucellosis, HSV, CMV, EBV, HIV, and others. Autoantibody screening was conducted for systemic autoimmune diseases (e.g. systemic lupus erythematosus, Sjogren’s syndrome, antiphospholipid antibody syndrome, Hashimoto thyroiditis), anti-neutrophil cytoplasmic antibody (ANCA) vasculitis and anti-GAD. Cerebrospinal fluid (CSF) analysis included cell count, protein levels, immunoglobulin G (IgG) index, PCR panel for viral infections, oligoclonal bands and all commercially available autoantibodies for autoimmune encephalitis. Electroencephalogram (EEG), cranial magnetic resonance imaging (MRI), and thorax–abdomen–pelvic computed tomography (TAP-CT) were performed for all patients. TAP-CT tumour screening was negative for all. FDG-18 positron emission tomography (PET) scans and brain angiography (CT/MRI) were performed as needed to aid diagnosis. The autoantibodies are listed in the supplementary information available at https://doi.org/10.1192/bjo.2025.10806.

The initial medical evaluation for catatonia before initiation of immunotherapy involved testing the following antibodies in CSF for all patients, except those with a clearly established alternative aetiology, such as previously diagnosed neurodegenerative diseases, glioblastoma multiforme, subarachnoid haemorrhage or lithium intoxication. The screening focused on autoimmune causes of catatonia, which were later confirmed as negative for all of the patients: NMDAR, α-amino-3-hydroxy-5-methyl-4-isoxazolepropionic receptor (GluR1 and 2), dipeptidyl-peptidase-like protein-6, leucine-rich glioma inactivated 1, contactin-associated protein-2, γ-aminobutyric acid receptor A and B, glycine receptor, IgLON-5, metabotropic glutamate receptor 1 or 5, and intracellular antigens (Hu, Yo, Ri, CV2 (CRMP5), amphiphysin, Ma1, Ma2, SOX1, Tr (DNER), Zic4, GAD65). All patients tested negative for viral agents in their CSF using a panel that included PCR tests for the following viruses: herpes simplex virus (HSV), Epstein–Barr virus (EBV), cytomegalovirus (CMV), varicella-zoster virus (VZV), human herpes virus (HHV) and influenza A or B.

The use of antipsychotic medications was limited within this sample. Any antipsychotic agents that were being taken by patients were discontinued upon admission to hospital. As recommended in catatonia guidelines, antipsychotic treatment was avoided during the in-patient period; however, in a limited number of cases presenting with severe agitation, quetiapine (100–300 mg per day) was initiated on an as-needed basis during hospital treatment. Comprehensive information regarding eaclinh patient’s prior use of antipsychotic and other psychotropic medications before referral to our clinic is provided in the supplementary material.

Response to steroids was retrieved from BFCRS scores and clinician-documented symptom resolution, including catatonia, psychosis and neurological signs. Maintenance immunotherapy was initiated in selected cases, with two patients receiving azathioprine and one receiving rituximab; the remaining patient received oral steroid treatments. The presence of severe catatonia symptoms and associated predominant cooperation difficulties during the initial evaluation prevented the administration of cognitive testing. Moreover, administering cognitive testing to patients experiencing severe catatonia and impaired consciousness would have compromised the relevance of the results. Follow-up assessments included the Montreal Cognitive Assessment and Frontal Assessment Battery at discharge and 6 months post-treatment, where available. Six months of follow-up assessments revealed that all patients were symptom-free.

Diagnostic criteria for autoimmune encephalitis, autoimmune psychosis, and antibody prevalence and encephalopathy score (APE2) ≥4, indicating immune involvement, were applied prospectively during hospital treatment and later analysed retrospectively. Patients were classified into possible autoimmune encephalitis, probable NMDAR encephalitis, and possible or probable autoimmune psychosis categories. In the retrospective analysis, we assessed how these frameworks supported clinical decision-making in real-world settings without definitive autoantibody confirmation. We also aimed to identify shared clinical and paraclinical characteristics among steroid-responsive patients rather than establishing statistical associations and explore the potential utility of these characteristics in guiding treatment decisions.

This study was approved by the Ankara University Faculty of Medicine ethical committee and clinical research board (2024000604-1/2024/604) and conducted according to the principles of the Declaration of Helsinki. Informed consent was obtained, but the requirement for written consent was waived owing to the retrospective and de-identified nature of the data.

## Results

Among 56 screened patients, ten (nine female, one male; aged 21–63 years) presented with subacute-onset catatonia, delirium-like features and psychotic symptoms, all improving with empirical steroid therapy. BFCRS scores ranged from 11 to 39. The most common psychotic symptoms were misidentification delusions (70%), including Capgras or Cotard-like delusions, followed by paranoid delusions (20%) and grandiosity (10%). A viral-like prodrome and dysautonomia were each observed in 50% of cases. All patients in the cohort presented with new-onset psychiatric symptoms persisting for less than 12 weeks before admission (range: 1–8 weeks; median: 4 weeks). Mixed-type catatonia (50%) was the most frequent presentation, followed by excited (30%) and stuporous (20%) subtypes. One patient had a postpartum onset. All cases had delirium-like features, and 20% exhibited focal neurological signs such as atypical parkinsonism, myoclonus and facial dyskinesia. Clinical characteristics are detailed in Table 1.

**Table 1:** Clinical and paraclinical characteristics of patients with steroid-responsive catatonia

Case	Age	Gender	Clinical presentation	Psychiatric history	Delusion type	CSF findings	Systemic antibodies	FDG-PET findings	Flu-like prodrome	BDZ response	MRI findings	EEG findings
1	44	F	Excited catatonia	–	Paranoid-Persecutory	30 WBC/mm^3^	Negative	N/A	Yes	Partial	Unremarkable	Normal, 9–10 Hz
2	23	F	Postpartum mixed-type	–	Capgras	0 WBC/mm^3^	Negative	N/A	No	Partial	Splenial restriction, periventricular enhancements	Beta rhythm, 10 µV
3	43	F	Stuporous catatonia	–	Capgras	20 WBC/mm^3^	Negative	Bilateral hypometabolism (except medial temporal lobes)	Yes	No response	Unremarkable	Normal, 9–10 Hz
4	24	F	Mixed-type catatonia	–	Grandiose	10 WBC/mm^3^	Negative	N/A	Yes	No response	Splenial diffusion restriction	Normal, 8–9 Hz
5	63	F	Excited catatonia	Late-onset sx, possible FTD (2 years)	Cotard	0 WBC/mm^3^	Anti-TPO (595 IU/mL)	Severe global hypometabolism including occipital and visual cortex	No	No response	Mild cortical atrophy	Diffuse slowing, 6 Hz
6	31	F	Excited catatonia	Schizoaffective (10 years)	Paranoid	5 WBC/mm^3^, CNS type 2 OCBs	Anti-TPO (>600 IU/mL), Anti-TG (1177 IU/mL), ANA 1/100	Severe global hypometabolism including occipital cortex	No	No response	Unremarkable	Normal, 11 Hz
7	58	F	Mixed catatonia and atypical parkinsonism	Major depression (36 years), treatment-resistant (2 years)	Cotard	Type 4 OCBs (mirrored)	Anti-TPO (189 IU/mL), Anti-TG (597 IU/mL)	Severe global hypometabolism including occipital cortex	No	No response	Mild cortical atrophy	Borderline slowing, 7 Hz
8	49	F	Stuporous catatonia	Schizoaffective disorder	Cotard + Capgras	Type 4 OCBs (mirrored)	Anti-SSA+	Normal metabolism	No	No response	Bifrontoparietal and periventricular demyelinating lesions	Normal, 11–12 Hz
9	21	M	Mixed catatonia and atypical parkinsonism	Bipolar disorder	Capgras	0 WBC/mm^3^	Negative	Normal metabolism	Yes	Partial	Unremarkable	Normal, 11 Hz
10	44	F	Mixed-type catatonia	–	Capgras	7 WBC/mm^3^, IgG Index ↑ (1.026), Type 4 OCBs (mirrored)	Negative	Left cortical hypometabolism (frontal, parietal, temporal)	Yes	Partial	Unremarkable	Normal, 11 Hz

CSF, cerebrospinal fluid; F, female; M, male; FDG-PET, FDG-18 positron emission tomography; MRI, magnetic resonance imaging; EEG, electroencephalogram; OCBs, oligoclonal bands; sx, schizophrenia; CNS, central nervous system; BDZ, benzodiazepine; FTD, frontotemporal dementia; WBC, white blood cell; IgG, immunoglobulin G; ANA, antinuclear antibody; anti-TG, anti-thyroglobulin antibody; anti-TPO, anti-thyroid peroxidase antibody; anti-SSA+, positive anti-Sjögren’s-syndrome-related antigen A antibody; –, no previous psychiatric history; N/A, test was not performed.

Pleocytosis (40%, *n* = 4) was the most common CSF abnormality, with one case also showing an elevated IgG index (10%). Oligoclonal bands were detected in 40% of patients (type II: 10%,; type IV: 30%). Overall, 50% (*n* = 5) had CSF findings indicative of CNS inflammation. Serum anti-thyroid antibodies were positive in 30% (*n* = 3) and anti-Sjögren’s-syndrome-related antigen A (anti-SSA) antibodies in 10% (*n* = 1).

FDG-PET scans, available for seven patients, showed non-specific but atypical cortical hypometabolism in 71.4% (*n* = 5 of 7). All tested patients were negative for limbic and paraneoplastic autoantibodies. Clinical and paraclinical data are detailed in Tables 1 and 2. Table 2 summarises the paraclinical features observed in the ten-patient cohort. Notably, only one patient (case 9) showed no abnormalities in the paraclinical investigations. However, this patient presented with a newly emerged subacute-onset neurological focal sign, unilateral parkinsonism, which itself is considered to be a clinical indicator suggestive of potential autoimmune involvement.

**Table 2:** Paraclinical findings, autoimmune diagnosis and APE2 scores in the ten-patient cohort

Paraclinical tests	Findings	Number of patients (%)
CSF analysis	Abnormal CSF findings indicating non-specific CNS inflammation	5 (50)
	Pleocytosis (WBC >5 per mm^3^)	4 (40)
	Elevated IgG index	1 (10)
	Type II oligoclonal bands (CNS-specific)	1 (10)
	Type IV oligoclonal bands (non-specific)	3 (30)
Autoimmune markers (serum)	Positive thyroid autoantibodies (anti-TPO, anti-TG)	3 (30)
	Positive anti-SSA antibodies	1 (10)
Brain imaging (MRI, FDG-PET, EEG)	Abnormal MRI findings reported by neuroradiologist as suggesting inflammation	3 (30)
	Splenium diffusion restriction	2 (20)
	Periventricular demyelinating or inflammatory lesions	2 (20)
	Cortical hypometabolism on FDG-PET	5 (of 7; 71.4)
EEG	Diffuse slowing	2 (20)
Overall paraclinical profile	Patients with no abnormal findings across CSF, imaging and EEG	1 (10)
Autoimmune encephalitis classification by Graus et al^6^	Patients remained classified as having possible autoimmune encephalitis	8 (80)
	Patients remained classified as having probable NMDA encephalitis	7 (70)
	Patients with possible autoimmune encephalitis also remained classified as having probable NMDAR encephalitis	5 (of 8; 62.5)
Autoimmune psychosis criteria by Pollak et al^20^	Patients fullfilling the criteria for probable autoimmune psychosis	4 (40)
APE2 scoring system	Patients with APE2 score ≥4 (indicating possible autoimmune aetiology)	8 (80)

APE2, antibody prevalence in epilepsy and encephalopathy; CFS, cerebrospinal fluid; CNS, central nervous system; MRI, magneticresonance imaging; FDG-PET, FDG-18 positron emission tomography; EEG, electroencephalography; NMDA, *N*-methyl-D-aspartate; NMDAR, *N*-methyl-D-aspartate receptor; IgG, immunoglobulin G; anti-TG, anti-thyroglobulin antibody; anti-TPO, anti-thyroid peroxidase antibody; anti-SSA, anti-Sjögren’s-syndrome-related antigen A antibody; WBC, white blood cell.

None of the patients met the Graus criteria for definite autoimmune encephalitis, probable seronegative autoimmune encephalitis or definite NMDAR encephalitis. However, 80% (*n* = 8) were classified as having possible autoimmune encephalitis, and 62.5% (*n* = 5 of 8) of these also met probable NMDAR encephalitis criteria. In addition, 20% (*n* = 2 of 10) met probable NMDAR encephalitis criteria but did not fulfil possible autoimmune encephalitis criteria, bringing the total number of probable NMDAR encephalitis cases to 70% (*n* = 7). All patients met possible autoimmune psychosis criteria, and 40% (*n* = 4) also met probable autoimmune psychosis criteria; 80% (*n* = 8) had an APE2 score ≥4, suggesting possible autoimmunity, but two patients with a score of 2 still responded well to steroids, indicating that the predictive value of APE2 is limited. The final autoimmune diagnoses are detailed in Table 2, and individual diagnostic classifications are shown in Table 3.

**Table 3:** Patients’ final diagnostic classifications, APE2 scores and key paraclinical findings for initiating empirical immunotherapy

Cases	Possible autoimmune encephalitis (Graus criteria)^6^	Probable NMDAR (Graus criteria)^6^	Probable autoimmune psychosis by Pollak et al^20^	Possible autoimmune psychosis	APE2 score (≥4, suggesting autoimmune encephalitis)	SREAT suspected	Key paraclinical findings leading to initiation of empirical immunotherapy
Case 1	+	+	+	+	6	X	Pleocytosis (30 WBC/mm^3^)
Case 2	+	X	X	+	7	X	Splenium diffusion restriction, periventricular radial dotted intensities with contrast enhancement on MRI, exclusion of infectious aetiologies
Case 3	+	+	+	+	6	X	Pleocytosis (20 WBC/mm^3^), cortical hypometabolism on FDG-PET
Case 4	+	+	+	+	7	X	Pleocytosis (10 WBC/mm^3^), splenium diffusion restriction on MRI, exclusion of infectious aetiologies
Case 5	X	+	X	+	2	+	Severe hypometabolism on FDG-PET, positive anti-TPO antibodies, moderate diffuse slowing (6 Hz) on EEG
Case 6	X	+	X	+	2	+	Type II oligoclonal bands in CSF, severe FDG-PET hypometabolism, positive anti-TPO and anti-TG
Case 7	+	+	X	+	5	+	Type IV oligoclonal bands in CSF, severe FDG-PET hypometabolism, positive anti-TPO and anti-TG, borderline diffuse slowing (7 Hz) on EEG
Case 8	+	X	X	+	4	X	Anti-SSA+, demyelinating MRI lesions, exclusion of primary demyelinating diseases
Case 9	+	X	X	+	4	X	Neurological focal signs, no significant paraclinical findings
Case 10	+	+	+	+	7	X	Pleocytosis (7 WBC/mm^3^), elevated IgG index in CSF, cortical hypometabolism on FDG-PET

APE2, antibody prevalence in epilepsy and encephalopathy; NMDAR, *N*-methyl-D-aspartate receptor; SREAT, steroid-responsive encephalopathy associated with autoimmune thyroiditis; CSF, cerebrospinal fluid; FDG-PET, FDG-18 positron emission tomography; MRI, magnetic resonance imaging; EEG, electroencephalography; IgG, immunoglobulin G; anti-TG, anti-thyroglobulin antibody; anti-TPO, anti-thyroid peroxidase antibody; anti-SSA+, positive anti-Sjögren’s-syndrome-related antigen A antibody; WBC, white blood cell; +, diagnostic criteria fulfilled; X, diagnostic criteria not fulfilled.

Among the paraclinical findings, abnormal CSF results indicating indirect inflammation of CNS represented the most common feature in this cohort. Moreover, several paraclinical findings were associated with fulfilment of possible autoimmune encephalitis, probable NMDAR encephalitis, and probable autoimmune psychosis criteria, with pleocytosis (40%) being the most commonly observed paraclinical abnormality. MRI abnormalities (hyperintense signal on T2-weighted fluid-attenuated inversion recovery sequences in multifocal areas compatible with inflammation or demyelination) were present in 20% of the cohort, and EEG slowing was detected in 20%, although these findings were not specific for autoimmune encephalitis. Table 1 summarises the main clinical and paraclinical characteristics of the patients included in the study. Table 2 shows paraclinical characteristics found in patients with subacute-onset immunotherapy-responsive catatonia.

All patients received a trial of sublingual lorazepam (7.5–12.0 mg per day), with only partial responses noted in 40% of patients (*n* = 4 of 10). All patients (*n* = 10) received methylprednisolone 1 g per day i.v. pulse therapy for 5–7 days, resulting in complete remission of catatonic, delirium, psychotic symptoms, and neurological symptoms including atypical parkinsonism, myoclonus and facial dyskinesia. BFCRS scores were reduced to zero in all patients. Patients’ clinical and paraclinical features leading them to undergo empirical immunotherapy are shown in Table 4. Detailed descriptions of the ten cases are presented in the supplementary material.

**Table 4:** Summary of clinical presentations and paraclinical findings supporting an underlying immune-mediated condition in patients with steroid-responsive catatonia

Case	Clinical presentation (prodrome and neuropsychiatric features)	Paraclinical findings and exclusion of other aetiologies
1	Viral-like prodrome; subacute catatonia with confusion, psychosis or possible autoimmune psychosis (+)	Pleocytosis (30 WBC/mm^3^); infectious aetiologies excluded
2	Viral-like prodrome; subacute catatonia with confusion, Capgras delusion, and dysautonomia or possible autoimmune psychosis (+)	Splenium diffusion restriction and periventricular contrast enhancements, radial hyperintensities on MRI; primer demyelinating and neurometabolic aetiologies excluded
3	Viral-like prodrome; subacute catatonia with Capgras delusion, confusion, and dysautonomia or possible autoimmune psychosis (+)	Pleocytosis (20 WBC/mm^3^), moderate atypical but non-specific global diffuse hypometabolism on FDG-PET; neurodegenerative and infectious aetiologies excluded
4	Viral-like prodrome; subacute catatonia with confusion, affective psychosis, and dysautonomia or possible autoimmune psychosis (+)	MRI: diffusion restriction; viral aetiologies excluded
5	Subacute catatonia with confusion and Cotard delusion evolving over 2 years of late-onset psychosis with treatment resistance or possible autoimmune psychosis (+)	Abnormal moderate diffuse slowing on EEG, anti-TPO (+), bilateral diffuse global hypometabolism including occipital and visual cortex; suspected SREAT; neurodegenerative diseases excluded
6	Subacute catatonia with confusion and paranoid delusions evolving over 10 years of treatment-resistant schizoaffective disorder or possible autoimmune psychosis (+)	Anti-TPO and anti-TG (+), type 2 oligoclonal bands in CSF; suspected SREAT
7	Subacute catatonia with confusion and Cotard-type delusions, dysautonomia, unilateral atypical parkinsonism, myoclonus, and facial dyskinesia or possible autoimmune psychosis (+)	Severe cortical hypometabolism on FDG-PET, positive anti-TPO and anti-TG; borderline EEG slowing, suspected SREAT, neurodegenerative, infectious and metabolic conditions excluded
8	Subacute catatonia with confusion, and Cotard–Capgras-type delusions or possible autoimmune psychosis (+)	Anti-SSA (+) with atypicaldemyelinating lesions on MRI; exclusion of primer demyelinating diseases
9	Viral-like prodrome, subacute catatonia with confusion and Capgras delusion, unilateral atypical parkinsonism or possible autoimmune psychosis (+)	No significant paraclinical abnormalities; clinical suspicion remained, remained classified as possible
10	Subacute catatonia with confusion and Capgras delusions and dysautonomia, evolving over years with fluctuating or possible autoimmune psychosis (+)	Pleocytosis (7 WBC/mm^3^), elevated IgG index in CSF, cortical hypometabolism on FDG-PET; infectious aetiologies excluded

SREAT, steroid-responsive encephalopathy associated with autoimmune thyroiditis; CSF, cerebrospinal fluid; FDG-PET, FDG-18 positron emission tomography; MRI, magnetic resonance imaging; EEG, electroencephalography; IgG, immunoglobulin G; anti-TG, anti-thyroglobulin antibody; anti-TPO, anti-thyroid peroxidase antibody; anti-SSA+, positive anti-Sjögren’s-syndrome-related antigen A antibody; WBC, white blood cell; (+) fulfilling the criteria for possible autoimmune psychosis.

## Discussion

In the present study, despite the absence of definitive autoantibody markers, we evaluated clinical and paraclinical indicators, response to immunotherapy, and potential underlying immune-mediated processes. By systematically reviewing patient records, we sought to highlight the importance of early immunotherapy in cases with suggestive but inconclusive autoimmune features and contribute to the ongoing discussion on autoimmune-related neuropsychiatric syndromes. Therefore, we aimed to analyse the most relevant clinical and paraclinical factors contributing to the decision to initiate early immunotherapy.

In this small cohort of ten patients, subacute-onset catatonia with delirious features was present in all cases (100%), suggesting a shared clinical pattern among those who responded to empirical immunotherapy. Psychotic symptoms were prominent, with Capgras or Cotard-like delusions being the most frequently observed. The most common form of catatonia in this cohort was mixed-type catatonia. Viral-like prodrome preceded symptom onset in 50% of the patients. Although autoimmune disorders are recognised to have a higher prevalence in females, the pronounced 9:1 female/male ratio observed in our sample could not be wholly explained by this epidemiological pattern. Instead, this disparity is more likely to be attributable to referral and selection biases shaping the composition of the studied clinical population.

Our findings highlight CSF abnormalities as the most frequent paraclinical indicator of CNS inflammation, with pleocytosis (40%), CNS-specific oligoclonal bands (10%) and elevated IgG index (10%) observed in subsets of patients. Notably, 50% of cases exhibited at least one inflammatory CSF marker, reinforcing the role of CSF analysis as a critical tool in the diagnostic workup of subacute catatonia patients. Pleocytosis in CSF was also the most frequently observed paraclinical abnormality and emerged as the most common marker fulfiling diagnostic criteria for possible autoimmune encephalitis, probable NMDAR encephalitis and probable autoimmune psychosis in our cohort, eventually leading to initiation of empirical immunotherapy. Given the high prevalence of inflammatory CSF markers in this cohort, CSF analysis should be prioritised in the diagnostic workup of catatonic patients.

Although none of the cases met the Graus criteria for definitive autoimmune encephalitis, a significant proportion fulfilled criteria for possible autoimmune encephalitis (80%) or probable NMDAR encephalitis (70%), suggesting an underlying immune-mediated process despite the absence of autoantibodies. In addition, all patients met possible autoimmune psychosis criteria, and most had APE2 scores ≥4, indicating potential autoimmune involvement. Notably, 20% met criteria for probable NMDAR encephalitis without fulfiling those for possible autoimmune encephalitis; this highlights differences between classification frameworks. According to the Graus algorithm, alternative diagnoses should be considered if possible autoimmune encephalitis criteria are not met. As our patients fulfilled possible autoimmune encephalitis criteria and showed a positive response to immunotherapy, an underlying autoimmune aetiology seemed highly likely. Their favourable acute-phase outcomes further supported this suspicion.

The majority of patients exhibited paraclinical markers of CNS inflammation, including pleocytosis, oligoclonal bands, elevated IgG index, MRI abnormalities, EEG slowing and cortical hypometabolism, findings that support the need for comprehensive autoimmune evaluations in suspected cases. However, relying solely on autoimmune encephalitis criteria may lead to missed diagnoses; thus, it is important to combine autoimmune encephalitis, autoimmune psychosis and APE2 criteria when evaluating patients with possible autoimmune catatonia. Although APE2 scores ≥4 are predictive of immune involvement, two (*n* = 2) low-scoring patients still responded well to steroids. Given that all patients in this study received steroids, we could not establish whether steroids were uniquely effective compared with conventional treatments. However, the findings strongly suggest that autoimmune psychosis criteria represent a valuable screening tool, that APE2 scores should be used alongside other frameworks rather than in isolation, and that paraclinical markers such as CSF pleocytosis, MRI abnormalities, EEG changes and PET hypometabolism may help to guide decisions regarding immunotherapy in patients with catatonia with delirious and psychotic features. In three cases, thyroid autoantibodies were detected alongside FDG-PET abnormalities, EEG slowing and type II oligoclonal bands, raising suspicion of steroid-responsive encephalopathy associated with autoimmune thyroiditis (SREAT). Owing to the variability in SREAT/Hashimoto’s encephalopathy criteria, thyroid autoantibodies were considered to be an epiphenomenon rather than a primary marker, and empirical steroids were initiated based on subacute-onset catatonia. Notably, all thyroid-antibody-positive patients in our cohort met probable NMDAR encephalitis or possible autoimmune encephalitis criteria, findings that further supported the use of empirical immunotherapy. Although the predictive value of autoimmune classification systems for steroid responsiveness remains uncertain, early steroid initiation may be beneficial once infections have been ruled out, as delays in treatment could lead to poorer prognosis.

Our study represents an example of the prospective application of several diagnostic criteria for patients with subacute-onset catatonia during the in-patient period, including the Graus criteria, APE2 criteria, and the autoimmune psychosis criteria proposed by Pollak et al. All patients improving with empiric immunotherapy fulfilled at least one of these autoimmune diagnostic criteria regarding the threshold for initiating empirical immunotherapy. Another important finding was that in our cohort, CSF examination had a major role in defining possible autoimmune encephalitis, probable NMDAR and probable autoimmune psychosis compared with MRI and EEG features.

Catatonia and its various forms, including catatonia with delirious features such as catatonic delirium or delirious mania, have been reported to be significant indicators of underlying autoimmune conditions, primarily anti-NMDAR encephalitis.^2,5^ As suggested in a catatonia consensus paper,^2^ people who develop catatonia after a short period of time should receive a full medical evaluation. This should include testing for autoantibodies in the serum, autoantibodies in the CSF, and inflammatory markers of autoimmune encephalitis and infectious agents, as well as EEG and MRI. Here, we presented ten cases of catatonia with clinical features suggesting a possible autoimmune aetiology. Autoantibody tests for limbic and paraneoplastic autoimmune encephalitis were conducted, and steroid treatment was initiated. All ten patients showed complete improvement following i.v. 1 g per day methylprednisolone pulse therapy, indicating potential autoimmune involvement. Despite the clinical improvement, autoantibody tests were negative. According to Graus’s diagnostic algorithm, none of the patients met the criteria for definitive autoimmune encephalitis, limbic encephalitis or seronegative probable autoimmune encephalitis.^6^

The paraclinical findings across the ten cases revealed various non-specific but suggestive indicators for CNS autoimmunity, such as pleocytosis (cases 1, 3, 4 and 10), elevated IgG index (case 10), cortical hypometabolism (cases 3, 5, 6, 7 and 10), structural changes including splenium lesions (cases 2 and 4) and demyelinating signals (cases 2 and 8), CNS-specific type 2 oligoclonal bands (case 6), non-specific mirrored type 4 bands (cases 7, 8 and 10), seropositive thyroid antibodies (cases 5, 6 and 7), anti-SSA antibodies (case 8) and non-specific EEG slowing (cases 5 and 7). Pleocytosis (cases 1, 3, 4 and 10) is a very important sign for diagnosis of autoimmune diseases because it often reflects an inflammatory process in the CNS but does not always indicate autoimmunity, especially in the absence of clear autoantibodies for CNS autoimmunity. Pleocytosis can also have other causes such as infection or cancer; therefore, it needs to be carefully interpreted together with other non-specific but suggestive signs of an autoimmune process. In our cases, additional indicators including atypical age at onset, subacute catatonic symptoms with altered consciousness, psychotic symptoms such as Capgras-like^7^ or Cotard-like delusions,^8^ failed response to lorazepam and electroconvulsive therapy, elevated IgG index, and non-specific but atypical cortical hypometabolism according to FDG-PET further suggested a potential autoimmune process.

Mild encephalitis/encephalopathy with a reversible splenial lesion (MERS) is a condition characterised by reversible abnormalities in the splenium of the corpus callosum on MRI.^9^ It can have various causes including infections, metabolic abnormalities, drugs and cerebrovascular diseases. MERS can cause neuropsychiatric symptoms such as confusion and agitation. Instances of delirious mania^10^ and catatonia^11^ have also been documented in cases of MERS. There is a lack of literature on cases of catatonia associated with MERS in the context of autoimmune causes. However, there is a possible connection between autoimmune processes and MERS, as indicated by the presence of specific autoantibodies such as anti-NMDAR autoantibodies.^12^ There were two cases of MERS in our cohort; in both cases (cases 2 and 4), the absence of other potential causes and the presence of suggestive indicators (pleocytosis in case 4 and atypical non-specific MRI findings including a splenial lesion in case 2), combined with atypical psychiatric manifestations such as subacute-onset catatonia, indicated a potential autoimmune or auto-inflammatory process.

Hashimoto’s encephalopathy, or SREAT, is often linked to seropositive thyroid antibodies, which may suggest an autoimmune aetiology but are not diagnostic on their own. They may support an autoimmune aetiology as an epiphenomenon.^13^ Combined with atypical clinical presentations and other non-specific but suggestive paraclinical evidence of CNS inflammation in our three cases with seropositive thyroid antibodies – such as severe cortical hypometabolism on FDG-PET, CNS-specific oligoclonal bands and moderate EEG background slowing – thyroid antibodies can support the possibility of an immune-mediated process (Case 5, 6, 7). Demyelination in imaging studies can be a sign of a number of autoimmune diseases, such as Sjögren’s syndrome with CNS involvement, which has been linked to neurological symptoms including paralysis, psychosis and catatonia.^14,15^ Despite the lack of definitive clinical and pathological markers for primary Sjögren’s, the presence of anti-SSA antibodies and MRI evidence of demyelination supported the likelihood of an autoimmune process in case 8.

Our patients’ diagnoses relied on clinical judgement owing to the absence of definitive biological markers for autoimmune encephalitis. This aligned with the criteria for diagnosing autoimmune catatonia in children and adolescents.^16,17^ The diagnostic algorithm emphasises a structured approach when no specific autoantibodies are detected in the CSF. Although our patients’ positive responses to steroids may suggest an autoimmune basis for their symptoms, this does not confirm an autoimmune condition without other supportive evidence. Dalmau and Graus have highlighted the risk of misdiagnosis and overdiagnosis of seronegative autoimmune encephalitis due to widespread use of autoantibody tests.^18^ Traditional reliance on antibody testing for autoimmune encephalitis diagnosis is problematic, as antibodies may not always be detectable.^7^ This poses a diagnostic challenge for patients without detectable autoantibodies in serum or CSF.

Patients presenting with catatonia, psychotic symptoms and altered consciousness may have underlying autoimmune conditions.^19^ Psychosis with catatonia is particularly indicative of an autoimmune process, akin to the concept of autoimmune psychosis.^20^ Steroid-responsive catatonia without a clear diagnosis of autoimmune encephalitis has been reported previously.^13^ In particular, there have been many cases in which catatonia has responded to steroids in the context of neuropsychiatric systemic lupus erythematosus.^21^ Among cases of paediatric autoimmune encephalitis, the presence of catatonia has also been found to predict response to steroids.^17^ However, steroids should not be viewed as an indiscriminate treatment for catatonia, particularly as steroids have occasionally been reported to induce catatonia.^22–24^ It is, therefore, more likely that steroids are an effective treatment in some cases of catatonia with a confirmed or suspected inflammatory aetiology.

Our case series highlighted the complexities of diagnosing catatonia with suspected autoimmune aetiologies. Steroid use was associated with improvement, even in cases without definitive autoimmune markers, demonstrating the importance of clinical judgement and comprehensive evaluation.^2^ This aligns with broader diagnostic methodologies advocating early immunotherapy to prevent neurological damage.^7^ As there is a delay between the onset of symptoms and antibody testing, it can be difficult to make a diagnosis. Therefore, it is important to be proactive about finding and treating possible autoimmune causes.

Our chart review showed that empirical steroids were initiated only in patients meeting at least one predefined autoimmune criterion (possible autoimmune encephalitis, probable NMDAR encephalitis, probable autoimmune psychosis, or APE2 ≥ 4), all of whom showed clinical improvement. This strong association between meeting autoimmune criteria and benefiting from immunotherapy suggests that autoimmune encephalitis, autoimmune psychosis, and APE2 frameworks can help to identify patients with catatonia that are likely to respond to treatment. Notably, inflammatory CSF markers emerged as key indicators for diagnosis and treatment initiation, further reinforcing the diagnostic value of autoimmune-related markers in guiding immunotherapy decisions.

Our findings suggest that structured diagnostic algorithms for autoimmune aetiologies can be clinically useful in catatonia cases, regardless of the exact autoimmune trigger. Patients meeting criteria for probable autoimmune psychosis, possible autoimmune encephalitis, probable NMDAR encephalitis, SREAT or APE2 ≥ 4 may be suitable candidates for empirical immunotherapy, especially if autoantibody tests are negative. However, reliance on a single algorithm may lead to treatable cases being overlooked, as seen in cases 5 and 6, in which the patients had low APE2 scores but met probable NMDAR encephalitis or suspected SREAT criteria. This underscores the need for a multiframework approach in treatment decisions.

The main limitation of this study was its small sample size, which restricts the generalisability of the findings and prevented the use of formal inferential statistics. The retrospective design introduced selection bias, as only steroid-treated patients were included. Although the findings were exploratory, they highlight the importance of considering immunotherapy in select cases, even when definite autoimmune encephalitis criteria are not met but clinical and paraclinical features suggest an autoimmune process. A comprehensive autoimmune workup in subacute-onset catatonia remains crucial for improving prognosis.

Psychiatrists should maintain a heightened awareness of autoimmune processes in patients with catatonia or with psychotic or delirium-like symptoms. Patients with autoimmune disorders can present with psychiatric symptoms, necessitating neuroimmunological assessments and treatments in psychiatric settings. The cases presented here suggest a link between autoimmune encephalopathy and catatonia, with strong responses to steroid treatment, supporting the inclusion of immunotherapies in the treatment of selected psychiatric patients.

## Supporting information

Ilhan et al. supplementary material Ilhan et al. supplementary material

## Data Availability

The data associated with this study are available from the corresponding author (R.S.I.) upon reasonable request.

## Supplementary material

The supplementary material is available online at https://doi.org/10.1192/bjo.2025.10806
