# Supplementary material for: Retrospective chart review of cases of steroid-responsive catatonia: exploring a potential autoimmune aetiology
Source: BJPsych Open. 2025 Aug 15;11(5):e182. doi: 10.1192/bjo.2025.10806 (PMC12451723; doi:10.1192/bjo.2025.10806)
Supplement: Ilhan et al. supplementary material [file S2056472425108065sup001.docx]

**Case 1**

A 44-year-old female patient was referred with a four-week history of gradually worsening psychotic symptoms following a one-week flu-like prodromal period. Initially, she exhibited paranoid delusions, pressured speech, insomnia, psychomotor agitation, wandering around aimlessly, nudism, aimless touching of people, and refusing to eat and drink. Despite starting antipsychotic therapy (IM Flupentixol depot with a total of 1 time and IM zuklopentiksol acuphase with a total of 3 times at intervals of 3 days) at an outpatient clinic, she showed no response.

Upon admission, the patient seemed inattentive and confused. Her medical and psychiatric history was unremarkable. There was no reported psychosocial stressor. Family history for psychiatric and autoimmune disease were unremarkable. Throughout the hospitalization period, the neurologic and psychiatric examination was notable for catalepsy, verbal and motor perseveration, disinhibition, agitation, impulsivity, motor excitation, and aimless aggression. She also displayed a grasp reflex, suggesting excited catatonia.

Additional symptoms included delusional thinking, pressured speech, short-term retrograde and anterograde episodic memory dysfunction, and fluctuating altered consciousness, which encompassed confusion and disorientation.  The Bush-Francis Catatonia Rating Scale (BFCRS) (Bush et al., 1996) was 11.  The Confusion Assessment Method (CAM) (Inouye et al., 1990) was positive for delirium. A sublingual lorazepam trial for excited catatonia was administered at 12 mg/day and had a partial response.

The MRI was unremarkable. Awake EEG recording was normal with a posterior background rhythm of 9-10 Hz and no interictal epileptiform discharges. A CSF examination showed pleocytosis (30 WBC per mm^3^) with a normal igG index without infectious agents ruled out on PCR The patient received methylprednisolone (MP) 1 g/day IV pulse therapy for five consecutive days. On the 5th day of treatment, neurologic and psychiatric signs and symptoms fully improved. BFCRS score reduced to 0, and subsequent CSF analyses revealed 0 cells. Oligoclonal bands and autoantibodies for limbic and paraneoplastic AE were later confirmed negative in the initial CSF taken before immunotherapy.  Maintenance treatment with 64 mg/day of MP, tapered over nine months, was undertaken. After three weeks of hospitalization, no cognitive impairment was found, as indicated by Montreal Cognitive Assessment (MOCA) and Frontal Assessment Battery (FAB) scores. Nine months after discharge, she remained in remission.

**Case 2**

A 23-year-old female patient, within the four-week postpartum period, was admitted to our emergency service with stuporous catatonia accompanied by autonomic instability. According to the family, one week after birth, the patient began exhibiting Capgras-like delusions, anterograde amnesia, psychomotor agitation, insomnia, and an alternation between excited and stuporous catatonic states, accompanied by confusion and disorientation. The patient's past medical and psychiatric history were unremarkable. No prior psychosocial stressor was evident.

Upon admission, diazepam 10 mg IV infusion was administered, resulting in partial improvement of catatonia symptoms. During the hospital stay, neurological and psychiatric tests showed that the patient was going through a range of symptoms, including stuporous and excited catatonia, mutism, verbal and motor perseveration, staring, motor stereotypies, negativism, posturing, psychomotor agitation, echopraxia, disinhibition, and mood lability, along with outbursts of inappropriate emotion and changes in their sense of where they were.

Physical examination revealed hypertension, 190/100 mmHg, and tachycardia, 140 bpm. BFCRS was 39. Treatment with Lorazepam (12 mg/day) sublingually and 7 sessions of electroconvulsive therapy (ECT) resulted in a partial response. CSF examination revealed 0 WBC per mm^3^  with a normal igG index without infectious agents. MRI revealed diffusion restriction at the splenium of the corpus callosum, mild irregular, nonspecific multifocal T2 hyperintensities, and atypical dotted contrast enhancements in the periventricular area. The patient's other tests were within normal ranges. Awake EEG recording was beta rhythm due to medicaton effect and no interictal epileptiform discharges.

The patient received MP 1 g/day IV pulse therapy for five consecutive days. Neurological and psychiatric signs and symptoms were fully improved, and the BFCRS score was reduced to 0. Subsequent MRI showed complete resolution of the splenial lesion while minimal dotted contrast enhancements in the left periventricular area persisted. Oligoclonal bands and autoantibodies for limbic and paraneoplastic AE were later confirmed negative in the initial CSF taken before immunotherapy. A maintenance treatment with 64 mg/day of MP tapered over six months, was initiated. No cognitive impairment was found after three weeks of hospitalization, as indicated by MOCA and FAB scores. The patient remained in remission for a 12-month follow-up period.

**Case 3**

A 43-year-old female patient was hospitalized due to a one-week history of stuporous catatonia, accompanied by autonomic instability and a clouding of consciousness. According to the family, the patient had exhibited Capgras-like delusions, paranoid delusions, olfactory and visual hallucinations, perseverative speech, disorientation, inattentiveness, aimless wandering at home, psychomotor excitation, aimless aggression, insomnia, and nudism over the course of a month. A one-week flu-like prodromal period and headache preceded the onset of neuropsychiatric symptoms.

The patient had no significant past medical or psychiatric history. There was no prior social stressor reported by the family. Upon admission and during hospitalization, neurological and psychiatric examination revealed the fluctuating course of stupor, rigidity, staring, echopraxia, posturing, mutism and grasp reflex suggestive of catatonia. Additionally, there was a fluctuating course of decreased consciousness, including inattentiveness, somnolence, disorientation, and confusion. A Physical examination revealed hypertension, 160 / 90 mmHg, and tachycardia, 105 bpm. The BFCRS score was 22.

A trial of lorazepam 12 mg/day was administered sublingually without any response. The CSF analysis revealed pleocytosis with 20 WBC per mm^3^, and a normal IgG index without infectious agents was ruled out on PCR. FDG-PET scan showed bilateral global hypometabolism, including bilateral occipital lobes.  Awake EEG recording was normal with a posterior background rhythm of 9-10 Hz and no interictal epileptiform discharges.

The patient received IV pulse therapy of 1 g/day of MP for five consecutive days, resulting in complete remission of neurological and psychiatric signs and symptoms by the third day of pulse therapy. BFCRS score was reduced to 0, and repeat CSF examination revealed 0 cells. Oligoclonal bands and autoantibodies for limbic and paraneoplastic AE were later confirmed negative in CSF.

Maintenance treatment with 64 mg/day of MP, tapered over six months, was undertaken. No cognitive impairment was found after three weeks of hospitalization, as indicated by MOCA and FAB scores. During the 6-month follow-up period, the patient did not exhibit any symptoms.

**Case 4**

A 24-year-old female patient was referred to our department for grandiose-religious delusions, agitation; pressured, perseverative and, disorganized speech; insomnia, disinhibition, aimless wandering, and an extended period of nudity, which developed six weeks before the hospital admission. A one-week flu-like prodromal period preceded the onset of neuropsychiatric symptoms. The patient had no significant past medical or psychiatric history. The family did not report any psychosocial stressors.

During admission and hospitalization, neurological and psychiatric examinations revealed a fluctuating course of verbal perseveration, grasp reflex, rigidity, staring, catalepsy, mutism, posturing, alternating with disinhibition, psychomotor excitation, agitation and impulsivity, emotional lability, suggestive of mixed catatonia encompassing stupor alternating with excitation. A fluctuation of decreased levels of consciousness was evident, accompanied by inattentiveness to the environment, disorientation, and confusion.

A physical examination revealed fluctuating tachycardia, alternating with bradycardia and hypertension.  The BFCRS score was 18. A trial of sublingual lorazepam 12 mg/day and six sessions of ECT with flumazenil 1 mg IV infusion were administered concomitantly without any response.

A CSF examination revealed 10 WBC per mm^3^ and a normal level of IgG index without infectous agents MRI showed ovoid diffusion restriction at the splenium of the corpus callosum without contrast enhancement. Awake EEG recording was normal with a posterior background rhythm of 8-9 Hz and no interictal epileptiform discharges.

IV pulse therapy of 1 g/d of MP was administered for five consecutive days, resulting in complete improvement of neurological and psychiatric signs and symptoms. The BFCRS was reduced to zero.  A further CSF examination revealed no cells.  The MRI showed complete resolution of the splenial lesion.

Maintenance treatment with 64 mg/day of MP, tapered over six months, was undertaken. After three weeks of hospitalization, no cognitive impairment was observed in MOCA and FAB performances upon discharge. During the 9-month follow-up period, the patient did not exhibit any symptoms.

**Case 5**

A 63-year-old female with a two-year history of treatment-resistant late-onset schizophrenia was hospitalized due to the symptoms of severe insomnia, aimless psychomotor agitation, wandering, verbal and physical aggression, and urinary incontinency that developed within two months. Her past psychiatric history revealed an insidious onset of nihilistic Cotard-like thoughts and reported personality change over the last two years, which included erratic behaviors, socially inappropriate behaviors such as inappropriate urination, hoarding, collecting foods from garbage, excessive smoking and eating, and an inability to perform daily living tasks. The patient's medical history documented six months of persistent constipation, which preceded the onset of psychiatric symptoms. Over the course of two years, the patient was treated with several antipsychotics, including paliperidone palmitate 100 mg/month IV for 8 months and risperidone po. at 8 mg/day for six months, but there was no significant improvement. The medical history was also notable for a diagnosis of autoimmune thyroiditis at the age of 33.

Upon admission to the clinic, the patient's medical history led to the suspicion of potential frontotemporal dementia instead of the diagnosis of treatment-resistant late-onset schizophrenia. Both neurologic and psychiatric evaluations identified various catatonic symptoms, including purposeless aggression, lack of inhibition, automatic compliance, grasping reflex, abnormal body postures, fixed gaze, impulsive behavior, excessive motor activity, and repetitive speech and movements. Additionally, the evaluations noted the presence of nihilistic delusions, speech derailment, orobuccal dyskinesia, and ideomotor apraxia. The BFRS was 19. Olanzapine, po 10 mg/day, and a trial of sublingual lorazepam, 7.5 mg/day, were initiated. The treatment was started without a discernible response.

A CSF analysis revealed no cells and a normal IgG index without infectious markers. Systemic autoimmune and vasculitis markers were negative except for anti-TPO (normal ranges: 0-34 IU/mL) positivity (595 IU/mL). The thyroid function test was within normal limits. A brain MRI revealed nonspecific global mild cortical atrophy. In the temporal and frontal lobes, there was no evidence of an atrophy pattern suggesting FTD. Awake EEG recording showed abnormal diffuse slowing with a posterior background rhythm of 6 Hz and no interictal epileptiform discharges. The FDG-PET scan revealed diffuse bilateral global cortical hypometabolism including occipital lobes and primary visual cortex, with no resemblance to any specific type of dementia syndrome. IV pulse therapy of 1 g/d of MP was administered for five consecutive days, resulting in complete improvement of catatonic symptoms by the 5th day. The BFCRS score was zero. Oligoclonal bands and autoantibodies for AEs were later confirmed negative in CSF.

**Case 6**

A 31-year-old female was hospitalized with acute disorientation, verbal perseveration, disinhibition, severe aggression, paranoid delusions, insomnia, and psychomotor agitation. The patient's psychiatric history revealed relapsing and partially remitting episodes for the last ten years. The episodes had progressively worsened, with incomplete remissions despite treatment attempts with numerous antipsychotics, mood stabilizers, and ECT. The patient was diagnosed with bipolar disorder and schizoaffective disorder by various psychiatric clinics.

Her medical history was remarkable for autoimmune thyroiditis, diagnosed at the age of 20. The patient’s family history was remarkable for autoimmune thyroiditis and autoimmune rheumatological diseases in the mother and the mother’s sister. There was no reported psychiatric history in the family.

During hospitalization, a neurologic and psychiatric examination revealed dysdiadochokinesia, dysarthria, ideomotor apraxia, bradykinesia, distractibility, alternated consciousness encompassing confusion, and disorientation. Catatonic signs with prominent delusional symptoms were evident. Catatonic symptoms consisted of impulsivity, disinhibition, combativeness, verbal and motor perseverations, unresponsiveness to environmental stimuli, rigidity, negativism, staring, and psychomotor agitation alternated with stupor. The BFCRS was 18. Quetiapine po 300 mg/ day was initiated for psychomotor agitation, and a trial of sublingual lorazepam 12 mg/day was administered, resulting in no improvement.

A CSF examination revealed five WBCs per mm^3^ and a normal IgG index without infectious agents. Serum anti-TPO was > 600 IU/mL(normal ranges 0-34 IU/mL) and anti-TG (normal ranges:0-115 IU/mL) was 1177 IU/mL. ANA was positive at 1/100 dilution. FDG-PET screening revealed significant bilateral diffuse global cortical hypometabolism, including bilateral occipital lobes. Awake EEG recording was normal with a posterior background rhythm of 11 Hz and no interictal epileptiform discharges. 5 days of 1 g/day steroid IV pulse therapy was initiated. Neuropsychiatric signs and symptoms improved completely on the third day of pulse treatment. The BFCRS was zero at the fifth day. Autoantibodies for limbic and paraneoplastic encephalitis were later confirmed as negative. CNS-specific type 2 oligoclonal bands were positive in CSF. The treatment plan involved gradually discontinuing quetiapine over the course of one month.

Maintenance treatment of MP at 64 mg/day with a tapered course and azathioprine at 100 mg/day for 9 months were undertaken. At discharge, no cognitive impairment was observed in FAB and MOCA tests performed at discharge. Throughout the 6-month follow-up period, the patient had no neuropsychiatric symptoms.

**Case 7**

A 58-year-old female patient was hospitalized following a suicide attempt with a history of treatment-resistant depression for two years. Past psychiatric history revealed a relapsing and remitting course of depressive episodes for 36 years, with recent exacerbations in severity and resistance to treatment over the last 2 years. The patient’s depressive episodes, characterized by marked psychomotor retardation, were initially episodic in nature. The patient initially exhibited a complete response to conventional antidepressant therapies within one week, which seemed atypical. Over the last two years, her depressive symptoms, characterized by severe psychomotor retardation, became markedly severe and unresponsive to multiple therapeutic interventions, which consisted of a variety of antidepressants, psychotherapy sessions, and ECT treatments. Her medical history revealed the diagnosis of autoimmune thyroiditis at the age of 30. Family history revealed that two daughters were diagnosed with autoimmune thyroiditis in their 20s without any reported history of psychiatric disorders.

By the third week of hospitalization, the patient developed an acute onset of behavioral and cognitive changes that were consistent with a delirium-like condition, characterized by a disturbance in attention and awareness that developed over a short period of time and fluctuated throughout the day, associated with disorientation, confusion, and visual hallucinations. Mixed-type catatonic symptoms were evident, including a fluctuating course of stupor and psychomotor agitation along with posturing, staring, and verbal perseveration. A neurologic and psychiatric examination revealed bilateral upper limb cogwheel rigidity and unilateral upper and lower limb resting tremor along with Cotard-like delusion and visual hallucinations. The BFCRS was 28. A trial of sublingual lorazepam 7.5 mg/day was initiated without response.

Serum anti-TPO was 189 IU/ml, and anti-TG was 597 IU/ml with normal thyroid function. The brain MRI examination was notable for nonspecific mild global cortical atrophy. Awake EEG recording showed nonspesific borderline slowing a posterior background rhythm of 7 Hz and no interictal epileptiform discharges. CSF analysis revealed no cells and a normal IgG index without infectious markers. The FDG-PET examination revealed severe bilateral diffuse cortical hypometabolism, including occipital lobes. MP IV 1 g/day pulse therapy was administered for five consecutive days. Neurologic and psychiatric signs and symptoms improved completely on the third day of pulse therapy. The BFCRS score was zero at the fifth day. Antibodies for limbic and paraneoplastic encephalitis were later confirmed as negative. The mirrored pattern type IV oligoclonal bands in CSF and serum were positive. A moderate level of executive dysfunction on MOCA and FAB was evident at discharge after two months of hospitalization. Maintenance treatment of MP at 64 mg/day with a tapered course and azathioprine at 100 mg/day for 9 months was undertaken. Throughout the six months of the follow-up period, moderate-level cognitive impairment persisted in MOCA and FAB performances, while improvements in psychosocial functioning and depressive signs were notable.

**Case 8**

A 49-year-old female patient with a one-year history of subacute onset psychotic symptoms, a fluctuating course of disorientation, and psychomotor agitation was hospitalized for subacute worsening of symptoms into Cotard-like delusions, psychomotor slowing and retardation, and constant disorientation and confusion within two months. Past psychiatric history revealed acute-onset insomnia, psychomotor agitation, confusion, Capgras-like delusions, paranoid delusions, extended periods of nudism, aimless wandering, inattention to the environment, staring periods during the day, and verbal and motor perseverations. According to the family, there were no known psychosocial stressors. The patient’s condition persisted despite various antipsychotic trials (risperidone 6 mg/day, olanzapine 20 mg/day, paliperidone palmitate 100 mg/month for 3 months), in addition to mood stabilizers (lithium and valproic acid). Her medical history was unremarkable for any neurological or autoimmune diseases.

Upon admission, all psychotropic medications were stopped. Neurologic and psychiatric examinations revealed catatonic signs, including verbal and motor perseveration, staring, grasping, negativism, posturing, and catalepsy, accompanied by confusion and disorientation. Bilateral bradykinesia, which affects both the upper and lower extremities, was also observed. The delusion of Cottard’s was evident. The BFCRS score was 15. A trial of sublingual Lorazepam 12 mg/day was administered without any particular response.

An extensive medical workup revealed positive serum anti-SSA +. An MRI examination revealed demyelinating lesions at periventricular, subcortical, and juxtacortical areas without contrast enhancement. Demyelinating lesions were inferred by neuroradiology to not to be compatible with any primary demyelinating disorders of CNS such as multiple sclerosis. Subsequent MRI and CT brain angiography screening were normal. Awake EEG recording was normal with a posterior background rhythm of 11-12 Hz and no interictal epileptiform discharges. A minor salivary gland biopsy revealed less than 50 lymphocytic cell infiltration, and Schirmer’s test was negative. CSF analyses revealed no cells and a normal IgG index without any infectious markers.

For seven consecutive days, the patient received MP 1 g/day IV pulse therapy. Neurological and psychiatric signs and symptoms improved on the 7^th^ day of pulse therapy. The BFCRS score was zero. Autoantibodies for limbic and paraneoplastic antibodies were later confirmed as negative. Mirrored pattern type IV oligoclonal bands were positive in both serum and CSF. No cognitive impairment was found after six weeks of hospitalization, as indicated by MOCA and FAB scores. Maintenance treatment with 32 mg/day of MP, tapered over six months, and azathioprine 100 mg/day for nine months were undertaken.

**Case 9**

A 21-year-old male patient was admitted with subacute-onset psychotic symptoms and psychomotor agitation. One weak flu-like period, according to the family, was followed by psychiatric symptoms such as insomnia, aimless psychomotor agitation, paranoid and Capgras delusions, aimless wandering, periods of staring and posturing, inattentiveness to the environment, and sudden aggression. The past psychiatric history revealed that the patient experienced the same symptoms two years ago, lasting two weeks without any particular treatment. There was no past medical or family history of psychiatric or medical diseases.

On admission, neurologic and psychiatric tests showed that the person was disoriented, had cogwheel rigidity in one upper limb, and bradykinesia in both upper limbs. Catatonic symptoms, such as staring, catalepsy, combativeness, and a fluctuating course of stupor, were evident. The BFRCS score was 13. A trial of 7.5 mg/day of sublingual lorazepam was initiated, with partial improvement in catatonic and psychotic signs. The BFRCS score was 3. There was no sign of Capgras’ delusion except the delusion of references. The patient continued to experience a fluctuating course of confusion and disorientation throughout the day, along with ideomotor apraxia and atypical parkinsonism signs such as unilateral upper extremity cog-wheel rigidity and bilateral bradykinesia in the upper extremities.

All laboratory and imaging investigations, including FDG-PET, were unremarkable for CNS inflammation. MP 1 g/day IV pulse therapy was administered for five consecutive days. On the third day of pulse therapy, neurologic and psychiatric signs resolved completely, as well as no cognitive dysfunction in MOCA or FAB performance was observed. The BFRCS was zero. Maintenance therapy was undertaken for 32 mg/d MP tapering for 2 months. The patient was symptom-free for 9 months.

**Case 10**

A 44-year-old female patient was hospitalized due to a one-week history of insomnia, psychomotor agitation, delusion of persecution, paranoia as well as Capgras delusions, and catatonic symptoms including staring, verbal and motor perseverations, disinhibition, and impulsivity. The family reported a one-week viral-like prodromal period preceding the neuropsychiatric symptomatology.

The patient's medical history revealed that he experienced an epileptic seizure eight years ago, following a one-week flu-like period eight years ago. There was no prior psychiatric history.

Upon hospitalization, psychiatric and neurologic examination revealed fluctuating signs of catatonia, such as staring, disinhibition, psychomotor excitation, stupor, and verbal perseveration. Paranoid delusions were notable. Disorientation and confusion were evident. The BFCRS was 13. The trial of sublingual lorazepam (7.5 mg/day) and olanzapine (10 mg/day) was initiated with partial responses in catatonia and psychomotor excitation.

The CSF examination was notable for 7 white blood cells per mm3 and an elevated IgG index (1,026) without infectious agents. The FDG-PET investigation revealed hypometabolism in the left frontal, parietal, and temporal cortex. Awake EEG recording was normal with a posterior background rhythm of 11 Hz and no interictal epileptiform discharges.

Infectious, metabolic, vascular, toxic, and paraneoplastic etiologies were excluded. For five consecutive days, the patient received MP 1 g/day IV pulse therapy. Neuropsychiatric signs and symptoms improved completely on the fifth day of pulse therapy, and the BFCRS score was reduced to 0. Mirrored-pattern OCBs type IV. Autoantibodies for limbic and paraneoplastic AE were later confirmed to be negative. Maintenance treatment with 64 mg/day of MP, tapered over six months, was initiated. Within one month after hospitalization, Olanzapine 10 mg/day treatments were stopped. No cognitive impairment was found after six weeks of hospitalization, as indicated by MOCA and FAB scores. The patient remained in remission for a 12-month follow-up period.
